# "I Feel like Exercising is a Waste of Time": A Convergent Mixed-Methods Study of Barriers to Physical Activity Among In-School Adolescents in Dar es Salaam, Tanzania

**DOI:** 10.34172/hpp.45890

**Published:** 2026-08-21

**Authors:** Zahra Abdi Aden, Olukayode Ganiu Odufuwa, Anita Mukarugaika Rugaika, Angel Dillip

**Affiliations:** ^1^School of Life Sciences and Bioengineering, The Nelson Mandela African Institution of Science and Technology, P.O. Box 447, Arusha, Tanzania; ^2^Vector Control Product Testing Unit (VCPTU), Ifakara Health Institute, Environmental Health and Ecological Sciences, P.O. Box 74, Bagamoyo, Tanzania; ^3^School of Materials, Energy, Water and Environmental Sciences, The Nelson Mandela African Institution of Science and Technology, P.O. Box 447, Arusha, Tanzania; ^4^Apotheker Consultancy (T) Limited, Health Access Initiative, Dar es Salaam, Tanzania

**Keywords:** Adolescent, Exercise, Health behavior, Health promotion, Mixed-methods, Qualitative research, Secondary schools, Tanzania

## Abstract

**Introduction:**

Only 3.9% of adolescents met the WHO-recommended physical activity level, highlighting the need to understand contextual barriers in urban Tanzania. Physical inactivity during adolescence contributes to future non-communicable disease risk, yet evidence on contextual barriers remains limited. Guided by a socio-ecological perspective, this study assessed physical activity among school-going adolescents and examined social and environmental influences.

**Methods:**

A convergent mixed-methods study was conducted in three public secondary schools in Kinondoni, Dar es Salaam, between July and November 2025. Quantitative data were collected from 381 adolescents aged 13-18 years selected through multistage and systematic sampling. Qualitative data were collected through three adolescent focus group discussions, three separate parent/guardian focus group discussions, and three physical education teacher interviews. Weekly physical activity was estimated from self-reported frequency and duration; at least 420 minutes/week was classified as meeting the WHO-recommended level. Quantitative data were analysed using univariable logistic regression, while qualitative data were analysed thematically.

**Results:**

Fifteen adolescents (3.9%) met the recommendation. Adolescents aged 16-18 years had lower odds of meeting the recommendation than those aged 13-15 years (OR 0.19, 95% CI 0.04-0.85), while boys had higher odds than girls (OR 8.85, 95% CI 1.97-39.78). Parental encouragement was inversely associated with meeting the recommendation (OR 0.28, 95% CI 0.10-0.83). Qualitative findings identified academic pressure, gender norms, social influence, limited facilities, and safety concerns.

**Conclusion:**

Interventions should address school, social, gender-related, and environmental barriers, particularly among girls and older adolescents.

## Introduction

 Adolescence is a critical stage for the development of health behaviors that often persist into adulthood.^1^ Regular physical activity (PA) during this period supports cardiorespiratory fitness, muscular and bone health, healthy body weight, mental well-being, and healthy development.^2,3^ The World Health Organization (WHO) recommends that children and adolescents aged 5 to 17 years achieve an average of at least 60 minutes per day of moderate-to-vigorous physical activity (MVPA) across the week, including vigorous activity and muscle- and bone-strengthening activity on at least three days per week.^4^

 Despite these benefits, insufficient PA remains common among adolescents globally.^5,6^ Global pooled evidence from 298 population-based surveys showed that 81% of school-going adolescents aged 11 to 17 years were insufficiently active in 2016.^6^ In Africa, a multi-country analysis of 23 countries found that only one in five in-school adolescents met the recommendation, with 25% of boys and 16% of girls achieving sufficient levels of PA.^7^ A broader analysis from 64 Global South countries reported the same pattern, showing that girls were consistently less active than boys across settings.^8^ In Tanzania, the 2022 WHO PA profile estimated physical inactivity among adolescents aged 11 to 17 years at 78% in boys and 86% in girls.^9^ These figures highlight a substantial public health challenge and suggest that low PA among adolescents remains a persistent concern. Understanding the factors that shape PA in specific urban contexts is therefore important.

 Physical inactivity during adolescence matters because it contributes to the wider non-communicable diseases (NCDs) burden.^10^ WHO identifies insufficient PA as a major behavioral risk factor for NCDs.^11^ In Tanzania, school-based evidence has shown that physical inactivity commonly occurs together with other NCD risk factors among in-school adolescents.^12^ This means adolescent PA behaviors should be viewed not only as a behavior in itself, but also as part of early NCD prevention.

 The school setting deserves particular attention because schools reach large numbers of adolescents each day and provide an important platform for health promotion.^13–15^ Public secondary schools are therefore a strategic setting for this study, particularly in Dar es Salaam, where increasing urban density and reduced access to safe community play spaces may leave school grounds as one of the few viable places for structured PA.^16,17^ Schools may also shape PA through timetables, supervision, available facilities, and the priority given to sport and exercise within everyday school life. Investigating barriers within this setting is therefore important for understanding why activity remains low and for identifying practical entry points for improvement.

 Dar es Salaam presents a strong case for focused study. It is a major urban city in Tanzania where adolescents are likely to experience many of the conditions associated with low PA. Yet the local evidence remains fragmented. Kinondoni district was selected among the five districts within the city because it is a densely populated urban municipality with a large adolescent population and marked socio-spatial diversity, including relatively affluent neighborhoods, commercially active mixed areas, and low-income informal settlements, making it a relevant setting for examining how social and environmental conditions shape adolescent PA.^18-20^

 Existing Tanzanian studies are stronger on obesity, nutrition, or broad school health challenges than on the everyday patterning of adolescent PA itself.^12,21^ The few studies that have examined PA directly among secondary school students in Kinondoni reported levels below the recommendation,^12^ but they did not combine prevalence estimates with qualitative accounts from adolescents, parents or guardians, and physical education teachers. This leaves an important local evidence gap on how measured PA levels align with the lived social, gender-related, school, and environmental barriers that shape adolescents’ opportunities to be active in urban public schools. This study therefore assessed PA engagement among secondary school adolescents in Dar es Salaam, using a threshold of at least 420 minutes per week to reflect the WHO recommendation of an average of at least 60 minutes per day multiplied by seven days, and examined factors associated with meeting this threshold while also exploring how parents or guardians and teachers influence adolescents’ PA.

## Methods

###  Study Design and Setting

 A convergent mixed-methods design, interpreted through a socio-ecological perspective, was used. Quantitative and qualitative data were collected over the same period and analysed separately. Integration was then conducted during interpretation through side-by-side comparison of quantitative patterns with qualitative themes and through a joint display. Quantitative findings on age, sex, class level, parental encouragement, awareness of NCD risk factors, and meeting the PA recommendation were compared with qualitative accounts from adolescents, parents or guardians, and PE teachers to identify areas of convergence, complementarity, and apparent divergence. The qualitative findings were used to explain and contextualize statistical patterns, including the low prevalence of meeting the recommendation, the gender difference in PA, lower activity among older adolescents, and the unexpected inverse association between parental encouragement and meeting the recommendation. The socio-ecological perspective guided interpretation across individual (age, sex, and individual awareness of non-communicable disease risks), interpersonal (peer encouragement and parental support), organizational or school (structural barriers), and community levels (broader structural landscape, including rapid urban density, unsafe neighborhood and recreational spaces). The study was conducted in Kinondoni Municipal Council in Dar es Salaam, Tanzania. Kinondoni had a population of 982,328 according to the 2022 Population and Housing Census and later population projections, and adolescents were estimated at 173,046, approximately 18% of the total population.^19^ The council secondary education profile reported 22 public and 57 non-government secondary schools in 2017, with 19,630 students enrolled in public secondary schools, including 9,574 boys and 10,056 girls.^20^ Data were collected from three public secondary schools between 25 July and 5 November 2025. School names are not reported to protect confidentiality.

 Public secondary schools were selected because they represent the major schooling context for adolescents in Kinondoni and provide an important setting for structured PA, particularly in urban areas where access to safe and well-maintained recreational spaces outside school may be limited.

###  Participants and Sampling

 The quantitative strand involved adolescents aged 13 to 18 years enrolled in Forms one to four across three public secondary schools in the Kinondoni Municipal Council. The minimum sample size was determined using a single population proportion formula and an estimated prevalence of 18% for meeting the WHO-recommended PA level. This 18% estimate represented the expected proportion meeting the WHO recommendation and was derived as the complement of the 82% inactivity prevalence reported in the WHO Tanzania PA profile. Since the primary outcome in this study was meeting, rather than not meeting, the WHO-recommended PA level, the complementary 18% proportion was used to align the sample size assumption with the outcome definition. Using 18% rather than 82% did not change the calculated sample size because the p(1-p) term is identical for complementary proportions. The calculation used a 95% confidence level, a 5% margin of error, a design effect of 1.5, and a 10% allowance for non-response. The calculated minimum sample was 379, and 381 consented adolescents were enrolled. Participants were selected through a multistage sampling procedure. First, three wards were randomly selected from the sampling frame provided by the Municipal Department of Education. Second, one public secondary school was randomly selected from each selected ward. Third, the required number of participants was allocated to each school in proportion to enrollment size and then stratified by form and sex. Within each stratum, students were selected using systematic random sampling from class registers.

 For the qualitative strand, purposive sampling was used to recruit adolescents, parents or guardians, and physical education teachers who could provide detailed perspectives on adolescent PA. Adolescent FGD participants were not drawn from the 381 survey respondents; rather, they were selected from eligible students who had not completed the survey. This approach was used to reduce respondent burden and to encourage open discussion that was not influenced by prior completion of the questionnaire. Participants were purposively selected to include both sexes and students from Forms one to four. Each adolescent FGD included eight students: four boys and four girls, with one boy and one girl selected from each of Forms one to four in each school. One FGD was conducted in each school, resulting in a total of 24 adolescent FGD participants. Parents or guardians of survey participants were recruited based on their availability and written informed consent. In addition, one physical education teacher with at least one year of teaching experience was recruited from each school for an in-depth interview. Overall, the qualitative sample comprised 47 participants: 24 adolescents in three FGDs, 20 parents or guardians in three FGDs, and three physical education teachers in three IDIs.

###  Data Collection and Measures

 Quantitative data were collected using a self-completed Kiswahili questionnaire, with research assistants available for clarification when needed. A mixed mode approach was used: the first school used Open Data Kit (ODK) Collect on Android tablets, whereas the remaining two schools used paper questionnaires due to logistical constraints. The paper forms were checked on site for completeness and later entered into ODK to create a single dataset. The questionnaire covered sociodemographic characteristics, awareness of selected NCD risk factors, PA frequency and duration, and selected social and school-related influences. Awareness items were adapted from a previous knowledge study,^22^ and PA questions were adapted from the Physical Activity Questionnaire for Adolescents (PAQ-A) manual.^23^ The tool was pretested with 20 adolescents from a nearby public secondary school that was not part of the final sample in order to improve clarity and feasibility without contaminating the selected study schools.

 Qualitative data were collected using semi-structured guides for adolescents, parents or guardians, and PE teachers. The guides explored the meaning of PA, perceived benefits, preferred activities, facilitators, barriers, and recommendations. All IDIs and FGDs were conducted in Kiswahili, audio recorded with permission and complemented by field notes.

 The primary outcome was meeting the WHO-recommended PA level. Weekly activity minutes were estimated by multiplying the midpoint of reported activity days per week (0, 1.5, 3.5, 5.5, or 7 days) by the midpoint of usual duration per active day (7.5, 22.5, 45.5, or 90 minutes). Adolescents with at least 420 estimated minutes per week were classified as meeting the recommendation. Although the questionnaire described physical activity as exercise that increased breathing rate and body warmth, intensity was not measured separately using a validated intensity measure; therefore, the outcome should be interpreted as an estimate of meeting the weekly PA recommendation and not as a direct measure of MVPA.

 Awareness of NCD risk factors were scored across 25 items and categorized using Bloom’s cut-offs: low (< = 59%), moderate (60-79%), and high (> = 80%), consistent with previous adolescent NCD knowledge research in East Africa.^24^ Examples of items included questions on whether NCDs could be transmitted or cured, which population groups were at risk, and whether family history, regular exercise, overweight, high sugar intake, alcohol use, salt intake, smoking, and dust exposure influenced NCD risk. The primary outcome was analyzed against the following variables: awareness of NCD risk factors, age group, sex, class level, socioeconomic status, parental encouragement, teacher encouragement, friends’ encouragement, participation in PE classes, usual mode of transport to school, and source of health information.

 The study protocol was reviewed and approved by an institutional review board before data collection. Written parental or guardian consent and written adolescent assent were obtained for adolescent participants, and written informed consent was obtained from adult participants.

###  Data Analysis

 Quantitative data were analyzed using Stata version 14.0. Participant characteristics were summarized using counts and percentages. Associations between each predictor and meeting the WHO-recommended physical activity level were assessed using univariable logistic regression, and these unadjusted results were treated as the primary quantitative findings. Because only 15 adolescents met the outcome, multivariable estimates were not used as confirmatory evidence. The de-identified quantitative dataset is provided as Supplementary file 1. A full exploratory multivariable model using 359 observations is provided in Supplementary file 2 and should be interpreted cautiously because of sparse outcome events and the possibility of overfitting.

 Qualitative data were audio recorded, transcribed verbatim in Kiswahili, translated into English, and managed in NVivo 14. The data were analyzed thematically using both deductive and inductive coding. Deductive codes were guided by the interview and discussion guides and the socio-ecological framework, while inductive codes captured themes emerging from participants’ views. Transcripts were read repeatedly for familiarization, after which codes were organized into broader categories and compared across participant groups and schools to develop the final themes. To improve coding consistency, two transcripts were recoded after two weeks, and discrepancies were discussed and used to refine the codebook. Findings were presented as thematic narratives supported by verbatim quotations.

## Results

###  Demographic and Socio-Economic Characteristics of Participants

 A total of 381 adolescents participated in the quantitative survey. Participants were relatively evenly distributed across the three schools and across class levels. More than half were aged 13-15 years (56.4%) and 55.9% were female. Most adolescents reported walking to school (64.3%). Quantitative participant characteristics are shown in Table 1, and qualitative participant characteristics are summarized separately in Table 2.

**Table 1:** Sociodemographic characteristics of quantitative participants (n = 381)

**Variable**	**Category**	**n (%) / Summary**
School	School 1	115 (30.2)
	School 2	146 (38.3)
	School 3	120 (31.5)
Age group (years)	13–15	215 (56.4)
	16–18	166 (43.6)
Sex	Male	168 (44.1)
	Female	213 (55.9)
Class level	Form 1	90 (23.6)
	Form 2	101 (26.5)
	Form 3	98 (25.7)
	Form 4	92 (24.2)
Mode of transport to school	Public transport	124 (32.6)
	Walk	245 (64.3)
	Other*	12 (3.1)
Socioeconomic status	Low	127 (33.3)
	Middle	127 (33.3)
	High	127 (33.3)
Mother’s education	No education	13 (3.4)
	Primary level	104 (27.3)
	Secondary or tertiary	167 (43.8)
	Do not know	97 (25.5)
Father’s education	No education	11 (2.9)
	Primary level	68 (17.9)
	Secondary or tertiary	186 (48.8)
	Do not know	116 (30.4)

Note: *Other transport included bicycle, motorcycle, tricycle and private car

**Table 2:** Summary characteristics of qualitative participants (n = 47)

**Participant group**	**Characteristic**	**n / Summary**
Adolescents	Focus group discussions	3
	Participants	24
	Sex	12 boys, 12 girls
	Age	13–18 years; mean 15.3
Parents/guardians	Focus group discussions	3
	Participants	20
	Sex	10 female, 10 male
	Age	27–63 years
Physical education teachers	In-depth interviews	3
	Participants	3
	Sex	3 males
	Age	28–47 years

###  Factors Associated With Meeting the WHO Recommended Physical Activity Level Among Adolescents

Table 3 shows that only 15 adolescents (3.9%) met the WHO-recommended PA level. In the univariable logistic regression, older adolescents aged 16-18 years had lower odds of meeting the recommendation than adolescents aged 13-15 years [1.2% (2/166) vs. 6.0% (13/215); odds ratio (OR): 0.19, 95% confidence interval (CI): 0.04-0.85]. Boys had higher odds of meeting the recommendation than girls [7.7% (13/168) vs. 0.9% (2/213); OR: 8.85, 95% CI: 1.97-39.78]. Class level was also associated with meeting the recommendation. Compared with Form 1 students [10.0% (9/90)], the odds of meeting the recommendation were lower among students in Form 2 [3.0% (3/101); OR: 0.28, 95% CI: 0.07-1.05], Form 3 [2.0% (2/98); OR: 0.19, 95% CI: 0.04-0.89], and Form 4 [1.1% (1/92); OR: 0.10, 95% CI: 0.01-0.80]. Parental encouragement was inversely associated with meeting the PA recommendation [9.4% (6/64) among those reporting no parental encouragement vs. 2.8% (9/316) among those reporting encouragement; OR: 0.28, 95% CI: 0.10-0.83]. Qualitative findings below help explain this apparent paradox. Mode of transport showed a pattern of lower activity among adolescents using public transport, but the estimates were imprecise; compared with the small group using other transport modes, walking was not associated with meeting the recommendation (OR: 0.62, 95% CI: 0.07-5.15), while the public transport estimate had a wide CI that crossed the null (OR: 0.09, 95% CI: 0.01-1.53).

**Table 3:** Factors associated with meeting the WHO recommended physical activity level among adolescents

**Variable**	**Category**	**Met WHO recommendation n/N (%)**	**Unadjusted OR (95% CI)**	***P*** **-value**
Awareness of NCD risk factors	Low	8/185 (4.3)	1.00 (ref)	—
	Moderate	6/163 (3.7)	0.85 (0.29–2.49)	0.761
	High	1/33 (3.0)	0.69 (0.08–5.72)	0.732
Age group (years)	13–15	13/215 (6.0)	1.00 (ref)	—
	16–18	2/166 (1.2)	0.19 (0.04–0.85)	0.030
Sex	Female	2/213 (0.9)	1.00 (ref)	—
	Male	13/168 (7.7)	8.85 (1.97–39.78)	0.004
Class level	Form 1	9/90 (10.0)	1.00 (ref)	—
	Form 2	3/101 (3.0)	0.28 (0.07–1.05)	0.059
	Form 3	2/98 (2.0)	0.19 (0.04–0.89)	0.036
	Form 4	1/92 (1.1)	0.10 (0.01–0.80)	0.030
Socioeconomic status	Low	5/127 (3.9)	1.00 (ref)	—
	Middle	7/127 (5.5)	1.42 (0.44–4.61)	0.556
	High	3/127 (2.4)	0.59 (0.14–2.52)	0.477
Parental encouragement	No	6/64 (9.4)	1.00 (ref)	—
	Yes	9/316 (2.8)	0.28 (0.10–0.83)	0.021
Teacher encouragement	No	3/49 (6.1)	1.00 (ref)	—
	Yes	12/329 (3.6)	0.58 (0.16–2.13)	0.413
Friends’ encouragement	No	2/94 (2.1)	1.00 (ref)	—
	Yes	12/269 (4.5)	2.15 (0.47–9.78)	0.323
Attends PE classes	No	2/31 (6.5)	1.00 (ref)	—
	Yes	12/342 (3.5)	0.53 (0.11–2.47)	0.417
	Don’t know	1/8 (12.5)	2.07 (0.16–26.22)	0.574
Transport to school	Other*	1/12 (8.3)	1.00 (ref)	—
	Walk	13/245 (5.3)	0.62 (0.07–5.15)	0.655
	Public transport	1/124 (0.8)	0.09 (0.01–1.53)	0.096
Source of health information	None/Other	1/18 (5.6)	1.00 (ref)	—
	Traditional	8/238 (3.4)	0.59 (0.07–5.01)	0.630
	Modern	6/125 (4.8)	0.86 (0.10–7.56)	0.890

Abbreviations: WHO, World Health Organization; NCD, non-communicable disease; PE, physical education; OR, odds ratio; CI, confidence interval; ref, reference category. Table 3 presents unadjusted associations only. Denominators vary slightly because of missing responses

###  Qualitative Results

 Qualitative findings from FGDs with adolescents and parents/guardians, and IDIs with PE teachers, are organized into six themes: (1) meaning and perceived benefits of PA, (2) enjoyment and preferred activities, (3) gender norms, body image, and comfort, (4) influence of peers, parents, and teachers, (5) academic pressure and competing routines, and (6) safety, space, and access to facilities. A joint display integrating quantitative and qualitative findings is presented in Table 4.

**Table 4:** A joint display integrating quantitative and qualitative findings

**Quantitative finding**	**Related qualitative theme**	**Integrated interpretation**	**Illustrative quote**
Very few adolescents met the weekly PA recommendation (3.9%)	Academic pressure; safety, space, and access to facilities	Low PA reflected school, family, and environmental constraints beyond individual knowledge or motivation.	*“Sports periods should truly exist, not only on paper.” (FGD 1, male adolescent, 16 years)*
Boys had higher odds of meeting the recommendation than girls	Gender norms, body image, and comfort	Girls' participation was constrained by embarrassment, social judgement, clothing concerns, lack of privacy, and gendered expectations.	*“For girls, the big field is often taken by boys. We are told to wait or use a corner. That reduces motivation.” (FGD 2, female adolescent, 15 years)*
Older adolescents had lower odds of meeting the recommendation	Academic pressure and competing routines	Examination pressure, homework, tuition, fatigue, household responsibilities, and phone use may reduce PA as adolescents get older.	*“For Form Four, the pressure of studies is high. I feel like exercising is a waste of time.” (FGD 2, female adolescent, 17 years)*
Parental encouragement was inversely associated with meeting the recommendation	Influence of peers, parents, and teachers	The finding may reflect reverse causality or encouragement without practical support; verbal encouragement may coexist with restrictions related to safety, time, academic priorities, or acceptable spaces for PA.	*“At home they say it is good to do sports, but when they see I play too much they tell me to reduce and go back to studying. So you don’t know what to follow.” (FGD 3, male adolescent, 15 years)*
Awareness of NCD risk factors was not associated with meeting the recommendation	Meaning and perceived benefits of PA	Participants generally understood the benefits and value of PA, but awareness did not overcome structural and social barriers to regular activity.	*“Generally, students have a positive attitude in what they say, but in practice their actions depend on friends and the environment.” (IDI 1, male PE teacher, 28 years)*

####  Theme 1: Meaning And Perceived Benefits Of PA

 Across all participant groups, PA was described positively and was generally understood as movement that activates the body, raises heartbeat, causes sweating, and supports health. Adolescents mostly described PA in terms of what it does to the body and mind, such as improving stamina, making the body feel lighter and helping them feel refreshed. Parents used a broader definition that also included walking and routine household tasks, while teachers explained that students generally understood the value of PA even though this awareness did not always lead to regular participation.

 “*To me, physical activity means doing something that strengthens the body so that it can protect itself from diseases. You keep your body active, not just sitting. When you exercise, you increase stamina, the heart works well, and you feel lighter.” (FGD 2, male adolescent, 16 years)*

 “*When a child is at home, even washing clothes, scrubbing the floor, carrying a bucket of water, or going to the market and coming back carrying things in the hands, the whole body is working. When our children help with these things; they build strength without even realising it.” (FGD 2, male parent, 58 years)*

 “*Generally, students have a positive attitude in what they say, but in practice their actions depend on friends and the environment.” (IDI 1, male PE teacher, 28 years)*

 Participants perceived PA as beneficial for physical health, emotional well-being, stress relief, confidence, stamina, and improved concentration. Adolescents described exercise as helping them feel happy, calm, and mentally refreshed, especially during stressful school periods. Parents similarly viewed PA as a way of strengthening the body and mind, and several also described sports as keeping adolescents occupied and away from harmful behaviors. Teachers reinforced these views by describing PA as important for both health and mental attentiveness in school. Although these benefits were strongly perceived in the qualitative views, such perceptions did not necessarily translate into regular PA for adolescents.

####  Theme 2: Enjoyment and Preferred Activities

 Enjoyment, refreshment, and personal interest were important reasons for engaging in sports and exercise. Adolescents most often mentioned football, basketball, skipping rope, jogging, dance, and informal games. For many, the attraction of sport came from fun, freedom, and social connection, while for others it also included competition, recognition, and future opportunity. Parents similarly described PA as something that could be made enjoyable at home through walking, music, playful competition, and family routines. Teachers also described students’ motivations as varied, including competition, enjoyment, perceived health benefits, parental encouragement, and the use of sport as a meaningful alternative to idleness.

 “*I do it because I like it. When you play football, you develop talent, you feel free, and you forget classroom matters.” (FGD 3, male adolescent, 15 years)*

 “*We do exercise as a form of refreshment on weekends, on Saturday and Sunday. My younger sibling really likes swimming and dancing, so during free time we see if we can go to the beach or play music and dance.” (FGD 1, male guardian, 27 years)*

 “*Some really like sports because of competition and enjoyment. Others join because of the health benefits, and others because they are encouraged by parents to reduce weight or stay fit.” (IDI 2, male PE teacher, 47 years)*

 Clear preferences for certain activities were also evident. Football was the first thing adolescents thought of when talking about PA, while basketball, netball, dance, and skipping rope were also frequently mentioned. Some adolescents viewed informal or home-based activities as more realistic than structured sports, especially where facilities were limited. Several adolescents also linked sports participation with future opportunities, including talent development, school teams, and possible employment through sport. These accounts show that motivation was shaped not only by health awareness, but also by enjoyment, identity, aspiration, and the practical opportunities available to adolescents.

####  Theme 3: Gender Norms, Body Image, and Comfort

 Gender norms, body image concerns, and comfort in public spaces shaped adolescents’ willingness to participate in PA, particularly among girls. Girls described embarrassment, fear of being watched, and discomfort in open play areas or when wearing sports clothes. Football in particular was often described as a boys’ activity, and some adolescents reported that girls who participated in sport could be judged negatively. Parents also described cultural and religious expectations around modesty, domestic roles, and acceptable clothing, while teachers noted that girls often wanted more privacy and safer, more comfortable participation spaces.

 “*Someone can say that if a girl plays football, she is a tomboy. That makes you feel bad, and some girls stop completely.” (FGD 1, female adolescent, 14 years)*

 “*Some parents do not like a girl to wear sports clothes; they see it as shameful or as showing off. So, they decide that she should stop completely instead of finding a modest way for her to exercise.” (FGD 2, male parent, 52 years)*

 “*The girls say they need a safe place to change clothes where they will not be watched.” (IDI 2, male PE teacher, 47 years)*

 This suggests that participation in PA was not simply a matter of personal choice. Rather, adolescents’ comfort and confidence were shaped by how they expected to be seen by parents, peers, and the community. In the quantitative analysis, boys had higher odds of meeting the WHO recommended PA level than girls. The qualitative findings help explain this difference by showing how self-consciousness, social judgement, and gendered expectations can reduce girls’ comfort and confidence to participate in PA.

####  Theme 4: Influence Of Peers, Parents, And Teachers

 Peers, parents, and teachers could either support or restrict participation in sports and exercise. Adolescents said they were more active when friends invited them to games, exercised with them, or made physical activity feel enjoyable, but peers could also discourage participation through teasing or by normalizing sedentary habits. Parents were described as important gatekeepers because they controlled time, permission, safety, and access to equipment. Teachers saw themselves as motivators and role models, but they also described limits in what they could do without enough time, facilities, and material support.

 “*There are others who laugh when they see you going to exercise and say you are just showing off.” (FGD 2, male adolescent, 17 years)*

 “*At home my parents say it is good to do sports, but when they see I play too much they tell me to reduce and go back to studying. So, you do not know what to follow.” (FGD 3, male adolescent, 15 years)*

 “*My mother does not like me going out in the evening. She fears safety. So even if I want to go play, I am forced to stay at home.” (FGD 3, female adolescent, 15 years)*

 “*If you see that a child is doing well in sports, you can support them by buying sports shoes, a jersey, socks, and other things. That way the child already has the equipment and can take part.” (FGD 2, female parent, 48 years)*

 “*When I’m with students, I take part with them in different sports. You know, motivation has to start with you first. I usually lead by example, I go down to the field myself and run with them, and they see that it is possible.” (IDI 3, male PE teacher, 32 years)*

 At the same time, social support was not always positive. Some adolescents described parents as prioritizing academic work or safety over sport, while teachers noted that their efforts could only go so far without school support. In the quantitative analysis, adolescents who reported parental encouragement had lower odds of meeting the WHO recommended PA level than those who reported no parental encouragement. The qualitative findings suggest that this clear contradiction may reflect the complexity of parental influence, where encouragement may coexist with restrictions related to safety, time, academic priorities, or the conditions under which PA is considered acceptable.

####  Theme 5: Academic Pressure And Competing Routines

 Time related pressure was one of the clearest barriers across all participant groups. School schedules, homework, tuition, household chores, and fatigue reduced the time available for regular PA. Adolescents also described social media and prolonged phone use as competing demands, while parents explained that children often returned home tired and still had domestic or religious responsibilities to complete. Teachers added that even school-based opportunities for PA were affected by academic priorities, especially during examination periods when physical education lessons could be interrupted or pushed aside.

 “*Social media contributes to it. You find someone using their phone for a long time on TikTok, Instagram, and games, so there ends up being no time for exercise.” (FGD 1, male adolescent, 15 years)*

 “*For Form Four, the pressure of studies is high. I feel like exercising is a waste of time.” (FGD 2, female adolescent, 17 years)*

 “*Because the time he comes back from school is not ideal, he gets back at around seven in the evening. When he comes back, he skips rope… and even that is only occasionally, because his time does not allow it.” (FGD 3, female guardian, 35 years)*

 “*Outside PE time, it is very difficult for these children to exercise because they are constrained by studies.” (IDI 3, male PE teacher, 32 years)*

 These findings suggest that physical inactivity was also contributed by competing demands within adolescents’ daily routines, where schoolwork and other obligations often took priority over movement. This pattern was also reflected in the quantitative analysis, where older adolescents had lower odds of meeting recommended PA level than those aged 13–15 years. Together, the qualitative and quantitative findings indicate that growing academic pressure and increasingly crowded daily routines restrict opportunities for regular PA as adolescents get older.

####  Theme 6: Safety, Space, And Access To Facilities

 Participants described PA opportunities as being shaped by the availability of safe open spaces, adequate school facilities, and basic sports equipment. Adolescents spoke about limited and poorly maintained play areas, overcrowding, and lack of equipment, which reduced the range of activities they could do and sometimes discouraged participation altogether. Parents focused mainly on safety and supervision, especially for girls and for PA outside school hours. Teachers also highlighted weaknesses in sports infrastructure, including limited space, lack of changing rooms, shortages of first aid supplies, and inadequate budgets for sports activities.

 “*There are only a few playgrounds, and some are not well-maintained. Sometimes they are even used for meetings and gatherings.” (FGD 3, female adolescent, 15 years)*

 “*Safety is also a limitation. In the evening, some playgrounds are occupied by suspicious people, so we parents become afraid and decide that the child should stay at home.” (FGD 1, female parent, 36 years)*

 “*The infrastructure is there, but it has shortcomings. We have a field for activities, but it is not specifically for any one sport. There are no changing rooms… the budget is small.” (IDI 2, male PE teacher, 47 years)*

 Even when adolescents were willing to be active, the physical environment often limited what they could do. For adolescents, lack of equipment, poor maintenance, and competition over space reduced opportunities to take part in regular sport. For parents, concerns about unsafe or unsupervised spaces made them reluctant to allow adolescents, particularly girls, to go out for exercise. Teachers similarly felt constrained by limited resources and insufficient infrastructure to support a wider range of sports. Overall, opportunities for PA were shaped not only by personal motivation and family support but also by the safety, availability, and accessibility of supportive physical environments.

## Discussion

 This convergent mixed-methods study was conducted among in-school adolescents in three public secondary schools in Kinondoni District, Dar es Salaam. Adolescence is a critical period for establishing lifelong healthy behaviors.^1^ The school setting remains one of the few structured environments through which large numbers of adolescents can be reached.^15^ Interpreted through a socio-ecological lens, the study assessed both the level of PA engagement and the everyday social and environmental factors that shape it by combining a school-based survey of 381 adolescents with FGDs and IDIs involving adolescents, parents or guardians, and PE teachers. The findings support strengthening school health programs, protecting time for physical education, improving school sports and physical education, and advancing multisectoral action across health, education, local government, and urban planning to make regular PA more feasible for adolescents in Dar es Salaam.

 Only 3.9% of adolescents in this study met the WHO-recommended PA level. While self-reported data demand cautious interpretation, the very low prevalence still indicates an important public health concern. The estimate was substantially lower than the 2022 WHO profile (22% boys and 14% girls) for Tanzania.^9^ Possible explanations include differences in self-report instruments, the conversion of categorical responses to estimated weekly minutes, the absence of separate intensity measurement, and that this study was limited to public school adolescents in urban Kinondoni. Comparisons with the 2014 Tanzania Global School-based Student Health Survey also require caution because that survey used a standardized surveillance instrument, whereas the present study converted categorical frequency and duration responses into estimated weekly minutes.^25^ Earlier evidence from Kinondoni Municipal Council also suggested that adolescent sports participation was below recommended levels: Kalabamu et al. reported that 87.2% of secondary school students regularly participated in physical sports activities, but only 23.3% participated more than three times per week and 50.5% spent more than 30 minutes per sports session; the authors concluded that frequency and duration were low compared with the WHO recommendation.^12^ This difference also indicates that regular participation in any sports activity is not directly equivalent to meeting the full weekly PA recommendation. This is particularly relevant because a population-based study from 140 countries reported that the prevalence of four or more NCD risk factors among adolescents increased from 14.8% in 2003-2007 to 44.0% in 2013-2017, with physical inactivity identified as one of the most common contributors.^26^ The persistence of low PA despite national policy attention to adolescent health and NCD prevention suggests an implementation gap between policy commitments and adolescents’ everyday opportunities to be active.^27,28^

 Older adolescents (aged 16-18 years) were less likely to meet the WHO-recommended PA levels. This finding is important and is well explained by the qualitative data. Adolescents in the older age group repeatedly described examination pressure, tuition, homework, fatigue, household chores, and prolonged phone use as crowding PA out of daily life. These pressures were not described as occasional interruptions, but as routine features of everyday life that made PA feel secondary or unrealistic. Frömel et al.^29^ similarly found that academic pressure was negatively associated with adolescents’ PA during recess and after school, suggesting that school-related stress can reduce movement during the physical education classes where PA is supposed to take place. The role of screen-based leisure in this study is also plausible. Zablotsky et al.^30^ using nationally representative US teen data, found that adolescents with higher non-schoolwork screen time were more likely to report infrequent PA, irregular sleep routines, and poorer social and emotional support. Although the context differs, this pattern is relevant to the current findings, where adolescents described TikTok, Instagram, gaming, and general phone use as competing with time for movement. The combined picture suggests that low PA among older adolescents in this study was not simply an age effect but reflected intensifying academic and sedentary demands that made regular PA harder to sustain.

 Knowledge alone did not appear to be sufficient to support regular PA in this study. Although adolescents, parents or guardians, and teachers could describe the benefits of PA, this awareness did not translate into regular participation. The quantitative results showed the same pattern: nearly half of participants had low awareness of NCD risk factors (48.6%), 42.8% had moderate awareness, and only 8.7% had high awareness. In the analysis, neither moderate nor high awareness was associated with meeting the WHO-recommended PA level. This lack of gradient suggests that awareness was not the main factor distinguishing active from inactive adolescents in this study. Martins et al.^31^ similarly found that adolescent PA is shaped by multiple interacting influences, with knowledge representing only one part of a much larger behavioral process. Alliott et al.^32^ also showed that adolescents may understand the value of PA but still experience barriers linked to competing demands, relationships, and the wider context of everyday life. Interventions should therefore move from awareness campaigns alone to environmental restructuring, including scheduled active recess, protected and mandatory PE, provision of equipment, and safe spaces for girls and boys to participate comfortably.

 The role of social support was more complex than expected. In the analysis, parental encouragement was inversely associated with meeting the WHO-recommended PA level, while teacher and friends’ encouragement were not statistically significant. This should not be interpreted as evidence that social support is unimportant. Rather, it suggests that encouragement may have operated in a context where other barriers were stronger than verbal support alone. From a socio-ecological perspective, parental encouragement is one interpersonal influence, but its effect depends on whether adolescents also have the time, permission, facilities, equipment, safe spaces, and school support needed to translate encouragement into regular PA. A meta-analysis of 112 studies reported that positive parental influence was associated with higher PA among children and adolescents, whereas negative parental influences such as punishment and discouragement were not consistently associated with PA engagement.^33^ Likewise, Lin et al.^34^ found that social support was positively associated with adolescent PA, partly through self-efficacy. In the present study, encouragement may often have reflected concern rather than enablement: parents may have advised adolescents to exercise because they were already inactive, while still limiting outdoor play because of safety concerns, academic priorities, household chores, or worries about gender norms and reputation. The qualitative findings support this interpretation by showing that parents were both supporters and gatekeepers. They valued PA in principle, but their ability to support it was limited by the conditions surrounding adolescents’ daily lives. This may explain why parental encouragement may have appeared negatively associated with meeting the recommendation, and why peer and teacher encouragement, although visible in the qualitative data, was not sufficient to overcome school, household, and environmental barriers.

 School barriers were a major part of the explanation for low PA in this study. Adolescents and teachers described interrupted PE classes, limited sports facilities, overcrowded play areas, lack of changing rooms, and a generally low priority given to sport within the school routine. These findings help explain why positive attitudes towards PA did not translate into regular participation. They are also consistent with evidence showing that school context matters strongly for adolescent PA behavior. Wang et al.^35^ found that daily physical education, school-organized extracurricular activities, encouragement to use sports resources, and greater availability of facilities were positively associated with adolescent PA, while Morton et al.^36^ showed that school policies, facilities, and the social climate of the school environment can influence whether adolescents are active or sedentary. Similar evidence from Ethiopia reinforces this point in an East African context: Biadgilign et al.^37^ highlighted the importance of focused and regular physical education in schools, and Zeleke et al.^38^ found that attending schools with sports or playground facilities was associated with higher PA among adolescents. These findings suggest that schools are not only places where adolescents learn about health; they are also environments that can either normalize movement or make it difficult to sustain. In the present study, the problem was therefore not simply low motivation among adolescents, but weak institutional conditions for making PA a regular, protected, and feasible part of school life.

 Barriers outside school were equally important and further limited opportunities for PA engagement. Adolescents described few safe and well-maintained places to play, while parents worried about unsafe community open spaces, especially in the evenings and particularly for girls. These concerns are supported by previous research. Ding et al.^39^ found that neighborhood safety, access to recreational spaces, and supportive built environments were important correlates of PA among young people. In African settings, Adebusoye et al.^40^ reported that safety, access, and recreational infrastructure are important built-environment considerations for PA among children and adolescents. The present findings are also consistent with those of Kinsman et al.^41^ who showed that adolescent girls’ PA in South Africa was constrained by gender norms, limited infrastructure, and home and community barriers. In Dar es Salaam, rapid urban growth and uneven access to safe open spaces may restrict opportunities for PA outside school. This means that even adolescents who are motivated to be active may remain inactive when both schools and neighborhoods fail to provide safe, accessible, and acceptable opportunities for PA engagement.

 A major strength of this study is its convergent mixed-methods design, which enabled triangulation of quantitative findings with the lived experiences of adolescents, parents or guardians, and physical education teachers. This provided a richer understanding of the social and environmental influences on PA engagement than either approach could have offered alone. Several limitations should also be considered. PA was self-reported and may be affected by recall and social desirability bias. PA intensity was not measured separately, and the assumption that reported PA corresponded to moderate-to-vigorous intensity may have led to overestimation of true MVPA. At the same time, midpoint conversion of broad frequency and duration categories may have introduced measurement error and possible underestimation or overestimation of weekly minutes near the cutoff. For example, midpoint conversion may overestimate weekly minutes for adolescents reporting high frequency but shorter activity duration within a category, while underestimating weekly minutes for adolescents reporting lower frequency but longer activity duration within a category. Because the estimated weekly minutes were derived from broad categorical self-report responses, sensitivity analyses using alternative thresholds such as 378 and 462 minutes (± 10% of 420 minutes) were considered but may imply a level of precision that the data do not support; future studies using continuous or device-based PA measures should assess the robustness of findings across alternative thresholds. Only 15 adolescents met the WHO recommendation, which reduced statistical precision and made multivariable modeling unstable; therefore, adjusted estimates were considered exploratory only.

 The cross-sectional design also precludes causal inference, particularly for the inverse association observed between parental encouragement and meeting the recommendation. The parental encouragement variable was measured using a single binary question and did not capture the quality, frequency, or type of parental support. The study included only public secondary schools in Kinondoni, so findings may not be generalizable to private schools, rural areas, or out-of-school adolescents. All PE teachers interviewed were male, which limited the inclusion of female teacher perspectives on gender-related barriers. Data were collected between July and November, and seasonal variation and school calendars may have influenced opportunities for PA. Finally, because the quantitative and qualitative components were conducted concurrently, issues that emerged from the qualitative data could not be tested quantitatively within the same study. Future studies could use sequential mixed-methods designs and device-based measures such as accelerometers to validate self-reported PA and examine unexpected findings in greater depth.

## Conclusion

 Physical inactivity remains a substantial concern among secondary school adolescents in Dar es Salaam. In this study, very few adolescents met the recommended level of PA, despite generally positive views about its value. The findings show that adolescent PA is shaped by linked influences at the individual, interpersonal, school, and community levels, and that low participation reflects not only limited opportunity for movement but also wider social and structural barriers. Addressing this problem will, therefore, require more than awareness raising alone. It will require strengthened school health programs, better school sports and physical education, protected opportunities for routine PA, and urban environments that are safe, accessible, and responsive to adolescents’ needs.

## Competing Interests

 The authors declare that they have no competing interests regarding authorship and/or publication of this article.

## Data Availability Statement

 De-identified quantitative data are provided as Supplementary file 1. Qualitative transcripts are not publicly available because they contain potentially sensitive information from adolescent participants; qualitative materials may be made available from the corresponding author on reasonable request, subject to ethical and institutional approval.

## Ethical Approval

 Ethical approval was obtained from the Ifakara Health Institute Institutional Review Board (IHI/IRB/No: 03-2025). The study certificate was dated 15 February 2025, and the approval was valid until 14 February 2026. Administrative permission was obtained from the relevant regional, municipal, education and participating school authorities, including the Dar es Salaam Regional Administrative Secretary (EA.260/307/04G/134) and Kinondoni Municipal Council (HB.345/49). Permission to publish the manuscript was granted by the National Institute for Medical Research (NIMR), Ref. No. BD.242/437/01C/223, dated 12 May 2026, under ethical clearance No. NATHREC-NEW-2021-108. Written parental or guardian consent and adolescent assent were obtained for student participants, and written informed consent was obtained from adult participants.

## Intelligence Use Disclosure

 The authors used ChatGPT, an AI-assisted language tool, only to support grammar, clarity, formatting, and language flow during manuscript preparation. The authors reviewed and verified all AI-assisted edits and take full responsibility for the content, accuracy, and integrity of the manuscript. ChatGPT was not used for data collection, statistical analysis, coding of qualitative data, or generation of research findings.

## Supplementary Files

Supplementary file 1. De-identified quantitative dataset used for the analysis. The file contains anonymized school labels and excludes consent numbers and direct identifiers.

Supplementary file 2. Exploratory multivariable logistic regression model for meeting the WHO-recommended physical activity level.
