# Supplementary material for: "I Feel like Exercising is a Waste of Time": A Convergent Mixed-Methods Study of Barriers to Physical Activity Among In-School Adolescents in Dar es Salaam, Tanzania
Source: Health Promot Perspect. 2026 Aug 21;16(2):232–43. doi: 10.34172/hpp.45890 (PMC13723411; doi:10.34172/hpp.45890)
Supplement: Supplementary file 1 [file hpp-16-232-s002.pdf]

Aden et al, Health Promotion Perspectives 2026;16(2):232-S2
doi:10.34172/hpp.45890
https://hpp.tbzmed.ac.ir

The adjusted model included 359 of 381 participants; those with missing data for one or more covariates were excluded.

**Variable**	**Category**	**Adjusted OR (95% CI)**	**p-value**
Awareness of NCD risk factors	Low	1.00 (ref)	—
	Moderate	0.84 (0.23–3.08)	0.797
	High	0.59 (0.05–6.24)	0.658
Age group (years)	13–15	1.00 (ref)	—
	16–18	0.10 (0.02–0.59)	0.011
Sex	Female	1.00 (ref)	—
	Male	11.15 (2.05–60.68)	0.005
Socioeconomic status	Low	1.00 (ref)	—
	Middle	0.84 (0.20–3.45)	0.808
	High	0.40 (0.08–2.10)	0.280
Parental encouragement	No	1.00 (ref)	—
	Yes	0.10 (0.02–0.41)	0.001
Teacher encouragement	No	1.00 (ref)	—
	Yes	0.38 (0.07–2.02)	0.259
Friends' encouragement	No	1.00 (ref)	—
	Yes	2.69 (0.44–16.34)	0.282
Transport to school	Other*	1.00 (ref)	—
	Walk	0.28 (0.02–4.61)	0.371
	Public transport	0.03 (0.001–1.03)	0.052

Abbreviations: WHO, World Health Organization; NCD, non-communicable disease; OR, odds ratio; CI, confidence interval; ref, reference category.
Estimates are mutually adjusted for the variables shown in the table: awareness of NCD risk factors, age group, sex, socioeconomic status, parental encouragement, teacher encouragement, friends' encouragement, and transport to school.
*Other transport included bicycle, motorcycle, tricycle, and private car.
